# Supplementary material for: Resolving the full spectrum of human genome variation using Linked-Reads
Source: Genome Res. 2019 Apr;29(4):635–45. doi: 10.1101/gr.234443.118 (PMC6442396; doi:10.1101/gr.234443.118)
Supplement: Supplemental Material [file supp_gr.234443.118_Supplemental_longranger-2.2.zip › longranger-2.2.tar.bz2!/lr2.2-public/lib/stan/cmdstan/stan/doc/api/html/parser_8hpp_source.html]

Stan: src/stan/gm/parser.hpp Source File


|  |  |
| --- | --- |
|  | Stan  2.5.0  probability, sampling & optimization |


- Main Page
- Related Pages
- Namespaces
- Classes
- Files

- File List
- File Members

All Classes Namespaces Files Functions Variables Typedefs Enumerations Enumerator Friends Macros Pages

- src
- stan
- gm

parser.hpp

Go to the documentation of this file.

1 #ifndef STAN\_\_GM\_\_PARSER\_\_PARSER\_\_HPP

2 #define STAN\_\_GM\_\_PARSER\_\_PARSER\_\_HPP

3

4 #include <boost/lexical\_cast.hpp>

5 #include <boost/fusion/include/adapt\_struct.hpp>

6 #include <boost/fusion/include/std\_pair.hpp>

7 #include <boost/config/warning\_disable.hpp>

8 #include <boost/spirit/include/qi.hpp>

9 #include <boost/spirit/include/qi\_numeric.hpp>

10 #include <boost/spirit/include/phoenix\_core.hpp>

11 #include <boost/spirit/include/phoenix\_function.hpp>

12 #include <boost/spirit/include/phoenix\_fusion.hpp>

13 #include <boost/spirit/include/phoenix\_object.hpp>

14 #include <boost/spirit/include/phoenix\_operator.hpp>

15 #include <boost/spirit/include/phoenix\_stl.hpp>

16 #include <boost/spirit/include/support\_multi\_pass.hpp>

17 #include <boost/tuple/tuple.hpp>

18 #include <boost/variant/apply\_visitor.hpp>

19 #include <boost/variant/recursive\_variant.hpp>

20

21 #include <boost/spirit/include/version.hpp>

22 #include <boost/spirit/home/support/iterators/line\_pos\_iterator.hpp>

23

24 #include <cstddef>

25 #include <iomanip>

26 #include <iostream>

27 #include <istream>

28 #include <iterator>

29 #include <map>

30 #include <set>

31 #include <sstream>

32 #include <string>

33 #include <utility>

34 #include <vector>

35 #include <stdexcept>

36

37 #include <stan/gm/ast.hpp>

38

39 #include <stan/gm/grammars/program\_grammar.hpp>

40 #include <stan/gm/grammars/whitespace\_grammar.hpp>

41 #include <stan/gm/grammars/expression\_grammar.hpp>

42 #include <stan/gm/grammars/expression07\_grammar.hpp>

43 #include <stan/gm/grammars/statement\_grammar.hpp>

44 #include <stan/gm/grammars/var\_decls\_grammar.hpp>

45

46 namespace stan {

47

48  namespace gm {

49

50  bool is\_space(char c) {

51  return c == ' ' || c == '\n' || c == '\r' || c == '\t';

52  }

53

54  bool is\_nonempty(std::string& s) {

55  for (size\_t i = 0; i < s.size(); ++i)

56  if (!is\_space(s[i]))

57  return true;

58  return false;

59  }

60

61  inline bool parse(std::ostream\* output\_stream,

62  std::istream& input,

63  const std::string& filename,

64  const std::string& model\_name,

65  program& result) {

66

67  using boost::spirit::multi\_pass;

68  using boost::spirit::make\_default\_multi\_pass;

69  using std::istreambuf\_iterator;

70

71  using boost::spirit::qi::expectation\_failure;

72  using boost::spirit::qi::phrase\_parse;

73

74  using boost::phoenix::construct;

75  using boost::phoenix::val;

76

77  stan::gm::function\_signatures::reset\_sigs();

78

79  std::ostringstream buf;

80  buf << input.rdbuf();

81  std::string stan\_string = buf.str();

82

83  typedef std::string::const\_iterator input\_iterator;

84  typedef boost::spirit::line\_pos\_iterator<input\_iterator> lp\_iterator;

85

86  lp\_iterator fwd\_begin = lp\_iterator (stan\_string.begin());

87  lp\_iterator fwd\_end = lp\_iterator (stan\_string.end());

88

89  program\_grammar<lp\_iterator> prog\_grammar(model\_name);

90  whitespace\_grammar<lp\_iterator> whitesp\_grammar;

91

92  bool parse\_succeeded = false;

93  try {

94  parse\_succeeded = phrase\_parse(fwd\_begin,

95  fwd\_end,

96  prog\_grammar,

97  whitesp\_grammar,

98  result);

99  std::string diagnostics = prog\_grammar.error\_msgs\_.str();

100  if (output\_stream && is\_nonempty(diagnostics)) {

101  \*output\_stream << "DIAGNOSTIC(S) FROM PARSER:"

102  << std::endl

103  << diagnostics

104  << std::endl;

105  }

106  } catch (const expectation\_failure<lp\_iterator>& e) {

107

108  std::stringstream msg;

109  std::string diagnostics = prog\_grammar.error\_msgs\_.str();

110  if (output\_stream && is\_nonempty(diagnostics)) {

111  msg << "SYNTAX ERROR, MESSAGE(S) FROM PARSER:"

112  << std::endl

113  << diagnostics

114  << std::endl;

115  }

116

117  throw std::invalid\_argument(msg.str());

118

119  } catch (const std::runtime\_error& e) {

120  std::stringstream msg;

121  msg << "PROGRAM ERROR, MESSAGE(S) FROM PARSER:"

122  << std::endl

123  << prog\_grammar.error\_msgs\_.str()

124  << std::endl;

125

126  throw std::invalid\_argument(msg.str());

127  }

128

129  bool consumed\_all\_input = (fwd\_begin == fwd\_end);

130  bool success = parse\_succeeded && consumed\_all\_input;

131

132  if (!success) {

133

134  std::stringstream msg;

135  if (!parse\_succeeded)

136  msg << "PARSE FAILED." << std::endl;

137  if (!consumed\_all\_input) {

138  // get rest of program

139  std::basic\_stringstream<char> unparsed\_non\_ws;

140  unparsed\_non\_ws << boost::make\_iterator\_range(fwd\_begin, fwd\_end);

141  // get next two lines (if possible)

142  msg << "PARSING HALTED AT LINE "

143  << get\_line(fwd\_begin)

144  << std::endl

145  << "UNPARSED STAN PROGRAM: "

146  << std::endl

147  << unparsed\_non\_ws.str()

148  << std::endl;

149  }

150  msg << std::endl << prog\_grammar.error\_msgs\_.str() << std::endl;

151  throw std::invalid\_argument(msg.str());

152  }

153  return true;

154  }

155

156

157  }

158 }

159

160 #endif

stan::gm::is\_space

bool is\_space(char c)

**Definition:** parser.hpp:50

program\_grammar.hpp

expression07\_grammar.hpp

expression\_grammar.hpp

stan::gm::parse

bool parse(std::ostream \*output\_stream, std::istream &input, const std::string &filename, const std::string &model\_name, program &result)

**Definition:** parser.hpp:61

stan::gm::function\_signatures::reset\_sigs

static void reset\_sigs()

**Definition:** ast\_def.cpp:138

statement\_grammar.hpp

stan::gm::program

**Definition:** ast.hpp:795

stan::gm::program\_grammar

**Definition:** program\_grammar.hpp:22

var\_decls\_grammar.hpp

ast.hpp

stan::math::e

double e()

Return the base of the natural logarithm.

**Definition:** constants.hpp:86

stan::gm::whitespace\_grammar

**Definition:** whitespace\_grammar.hpp:11

whitespace\_grammar.hpp

stan::gm::is\_nonempty

bool is\_nonempty(std::string &s)

**Definition:** parser.hpp:54


---

[ Stan Home Page ]

*© 2011–2014,
Stan Development Team.*
